# TOLKIN – Tree of Life Knowledge and Information Network: Filling a Gap for Collaborative Research in Biological Systematics

**DOI:** 10.1371/journal.pone.0039352

**Published:** 2012-06-18

**Authors:** Reed S. Beaman, Greg H. Traub, Christopher A. Dell, Nestor Santiago, Jin Koh, Nico Cellinese

**Affiliations:** 1 Florida Museum of Natural History, University of Florida, Gainesville, Florida, United States of America; 2 Interdisciplinary Center for Biotechnology Research, University of Florida, Gainesville, Florida, United States of America; American Museum of Natural History, United States of America

## Abstract

The development of biological informatics infrastructure capable of supporting growing data management and analysis environments is an increasing need within the systematics biology community. Although significant progress has been made in recent years on developing new algorithms and tools for analyzing and visualizing large phylogenetic data and trees, implementation of these resources is often carried out by bioinformatics experts, using one-off scripts. Therefore, a gap exists in providing data management support for a large set of non-technical users. The TOLKIN project (Tree of Life Knowledge and Information Network) addresses this need by supporting capabilities to manage, integrate, and provide public access to molecular, morphological, and biocollections data and research outcomes through a collaborative, web application. This data management framework allows aggregation and import of sequences, underlying documentation about their source, including vouchers, tissues, and DNA extraction. It combines features of LIMS and workflow environments by supporting management at the level of individual observations, sequences, and specimens, as well as assembly and versioning of data sets used in phylogenetic inference. As a web application, the system provides multi-user support that obviates current practices of sharing data sets as files or spreadsheets via email.

## Introduction

The increase in data intensive biology is evident in systematic biology as in any domain of biological research. New analytical methods, improved algorithms, environmental observing sensors, and an increasing need for data access, sharing, and re-purposing are driving fundamental changes in the way biological systematics is conducted, increasing the capacity to perform large scale analyses, often iterated over thousand of repetitions, and the ability to visualize the results at multiple scales.

Conventional wisdom has held that the physical sciences vastly out-scale the needs of biology for high performance computing, collaboration networks on the order of thousands of researchers, and sheer volume of data generated and analyzed. Physics collaborations like GryPhN (Grid Physics Network) and iVDGL (International Virtual Data Grid Network) and the OSG (Open Science Grid) have demonstrated success in sharing resources and expertise, often in the context of large instrument investments.

While collaborations within biological community may never reach the enormity of astrophysics, multi-lab collaborations are now commonplace in the systematics research community, driven in part through agency funding programs such as the National Science Foundation's Assembling the Tree of Life (ATOL) program.

Genomics has fueled a transformation into data-intensive, or data enabled science [1], [2], which can be originally attributed to the Human Genome Project [3], [4]. Where acquiring sequence data was previously a bottleneck, data produced through high throughput sequencing is doubling more quickly than our ability to carry out analyses, placing biology as an area of science currently pushing the tenets of Moore's Law [2], [5]. Similar trends exist in other areas of biological systematics. For example, digital imageries acquired to document observations are substantially increasing as well (e.g., MorphBank – www.morphbank.net, MorphoBank – www.morphobank.org, iDigBio – www.idigbio.org).

For every genome sequence produced, researchers will need to annotate, parse, and link this information back to the voucher specimen of the organisms under study, greatly increasing the overall complexity and challenge of integrating biocollection data with molecular data, cytology, morphology, ecology, images, and voucher specimens among many others. This broad data synthesis increases in complexity with the inclusion of more information resources such as climate, remote sensing, and geospatial data, where biologists are likely not the primary data producers nor the custodians.

As large public investments are often involved in the generation of data, stakeholders require those data to be made accessible and re-usable. Experiments need to be repeatable as well, through appropriate archiving of data sets, which, for example, is a goal of the Dryad project [6]. Emphasis on projects such as Dryad and metadata specifications such as the Ecological Metadata Language (EML) have typically been based on creating metadata records at the level of datasets to enable repeatability [7], [8]. While these are critical first-steps, addressing our abilities to re-use data remains problematic, simply because the focus needs to be at an atomic level of seamless data access inside individual data sets. Wieczorek et al. [9] noted that the lack of coordinated publishing and isolation between repositories in the biodiversity community creates obstacles toward integration and use. Parr et al. [10] provide a general review of evolutionary informatics addressing community progress with data sharing and highlighting the current shortcomings that still prevent data re-use and analysis repeatability.

**Figure 1:**
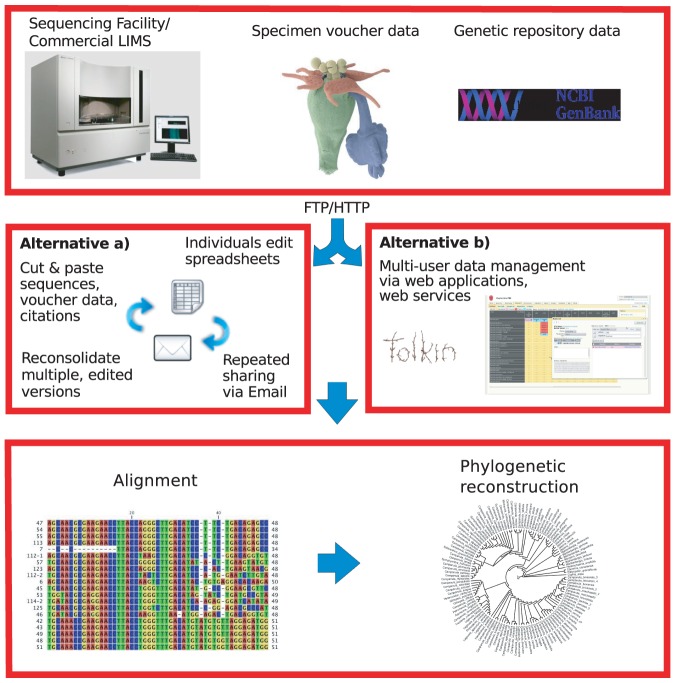
In a typical phylogenetic analysis workflow, common practice has been to manage data inside spreadsheets and in collaborative teams, to share them via email, as represented by Alternative a). While easy and effective for small data sets, spreadsheets can get out of sync and provenance is not well maintained. TOLKIN provides an Alternative b) to provide collaboration through a web portal, bulk data import and export of common formats, metadata and versioning support.

Many areas of biology, such as molecular systematics, are set to benefit substantially from the genomics revolution. Conversely, and particularly of significance to the domain of systematics, phylogenetic information is finding increased use in areas such as genetics, ecology, developmental biology and other organismal research. This leaves a critical need to manage and integrate data in systems that are interoperable, scalable, collaborative, and usable. Significant efforts have been invested in the development and deployment of Laboratory Information Management Systems (LIMS) aimed at documenting bench experiments and capturing direct results. LIMS are typically geared to specific research communities. In the organismic and evolutionary biology community, commercial systems such as Sequencher and Geneious address common needs targeting a large cross-section of researchers using molecular approaches and therefore, are feasible at the commercial level. Other LIMS are frequently aimed at clinical life science applications (e.g., STARLIMS).

**Figure 2:**
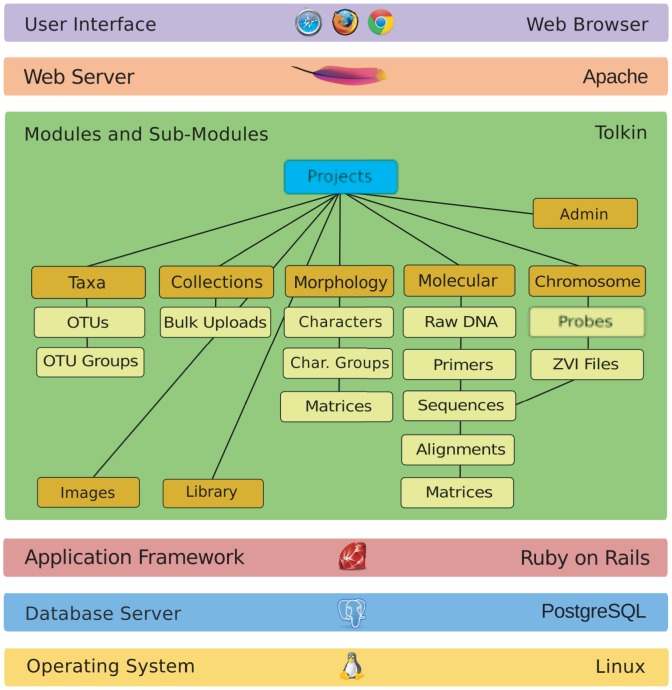
The TOLKIN architecture is built upon open source platforms and software, including Linux, Ruby on Rails and support for libraries, formats, and services such as BioRuby, NeXML, and GenBank. The diagram shows the relationship between TOLKIN modules and core data classes within each.

Less effort has been invested in the development of database environments that support the management of large collaborative datasets shared by smaller groups of researchers. We introduce the concept of a collaborative laboratory information management system (CLIMS). Figure 1 illustrates the gap that developed between the commercially viable LIMS, scripting efforts, and archival requirements for phylogenetic research.

A fundamental problem is that dataset size and complexity has increased to the point that managing and manipulating data is a challenge. Sharing data effectively through means such as spreadsheets is increasingly untenable. O'Leary and Kaufman [11] also noted that increased collaboration requires a shift from desktop, single-user systems to web-based multi-user support. Although software such as GBrowse, Geneious, and other tools are key to managing the deluge of genomic data, a need still exists to handle effectively large datasets that are based on either single marker sequences, combined genes, or morphological data, and include increasing numbers of OTUs and significant amount of metadata comprising biotic and abiotic information. Additionally, studies based on smaller scale sequencing are likely to continue as long as noise-free data can be generated quickly and inexpensively at local installations. Therefore, users need to have integrated access to data on the research collections that document their analyses, tissues, extractions, and integrated bibliographic data.

**Figure 3:**
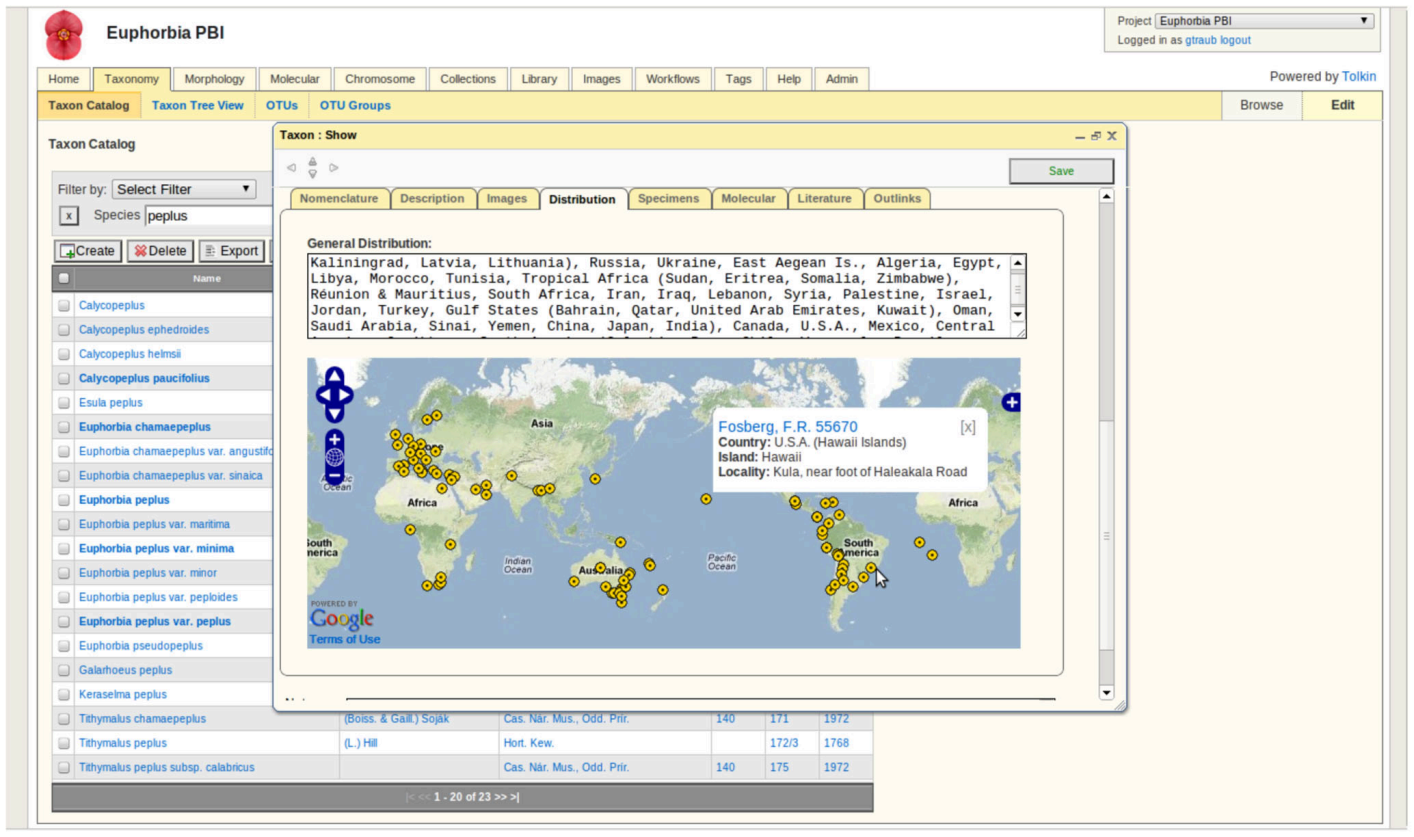
Taxonomy module. A taxon name is clicked in the taxon catalog to open a window containing a summary of its information stored across all modules. Additional tabs allow information to be displayed in a taxon tree view and OTUs and OTU groups to be created by the user.

TOLKIN – Tree of Life Knowledge and Information Network (www.tolkin.org) – is a collaborative web application designed to support research in biological systematics and other areas of biodiversity science. TOLKIN is domain agnostic and was designed to bridge identified gaps between commercial LIMS aimed primarily at data analysis or visualization, and the archiving of data in long-term repositories (e.g., GenBank). As a centralized resource, it emphasizes 1) web-based access to support long-distance collaboration; 2) data management for taxonomic information, molecular and morphological observations, biological collections and literature references; 3) capability to bulk import data from resources such as GenBank and to integrate with other services and stand-alone through common formats, including Nexus [12], NeXML [13], Darwin Core [9], and spreadsheets; 4) production of public taxon pages that are automatically generated and include data and images users wish to make available to the community; and 5) ease of use, flexibility in data sharing by getting a multi-user web application to behave as a desktop tool.

**Figure 4:**
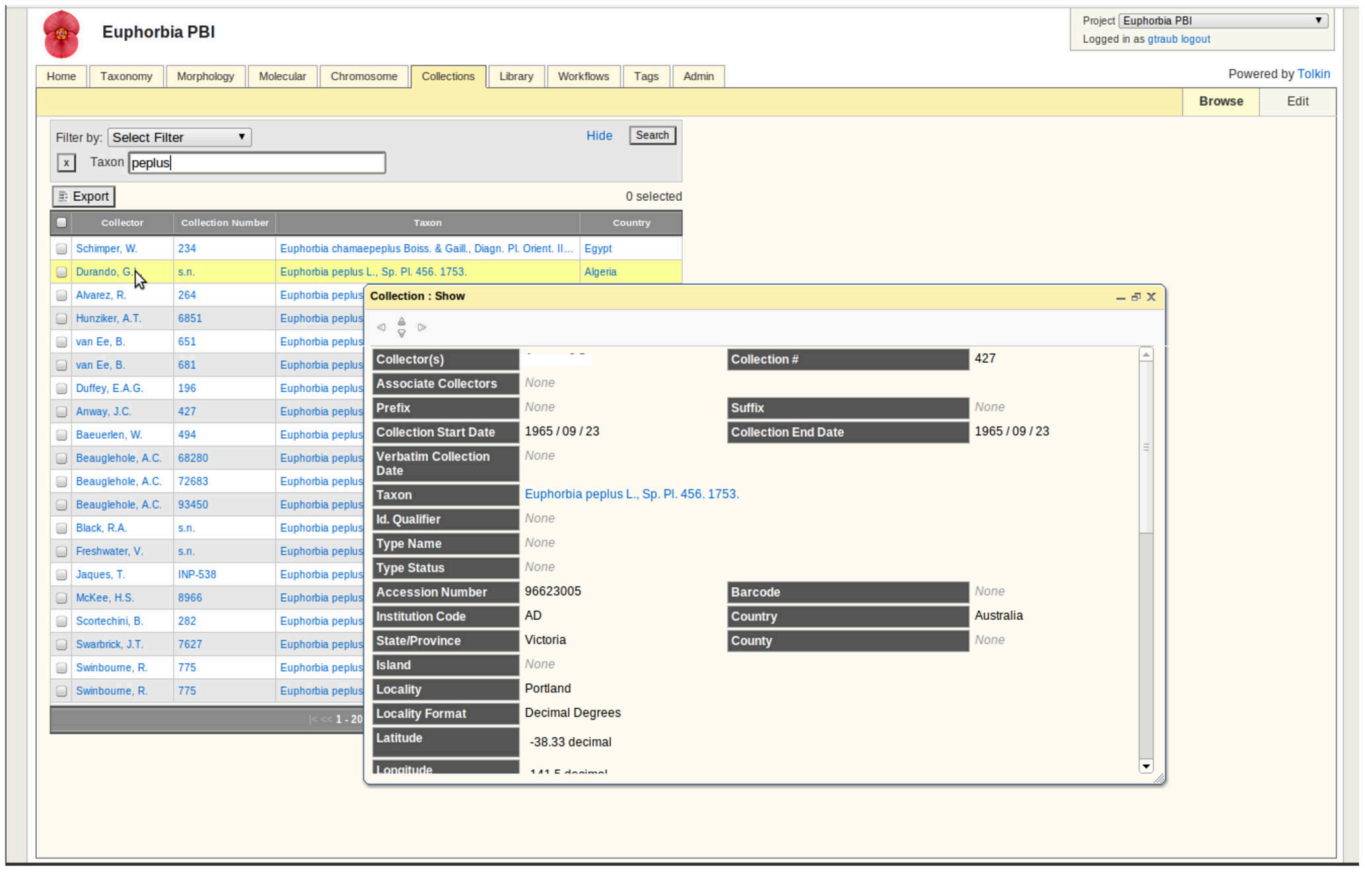
Collections module. This module stores information regarding biocollections. Searches can be filtered by selecting one or more parameters (e.g., taxon names, localities, etc.). Information is viewed by clicking on any item in the retrieved output list.

**Figure 5:**
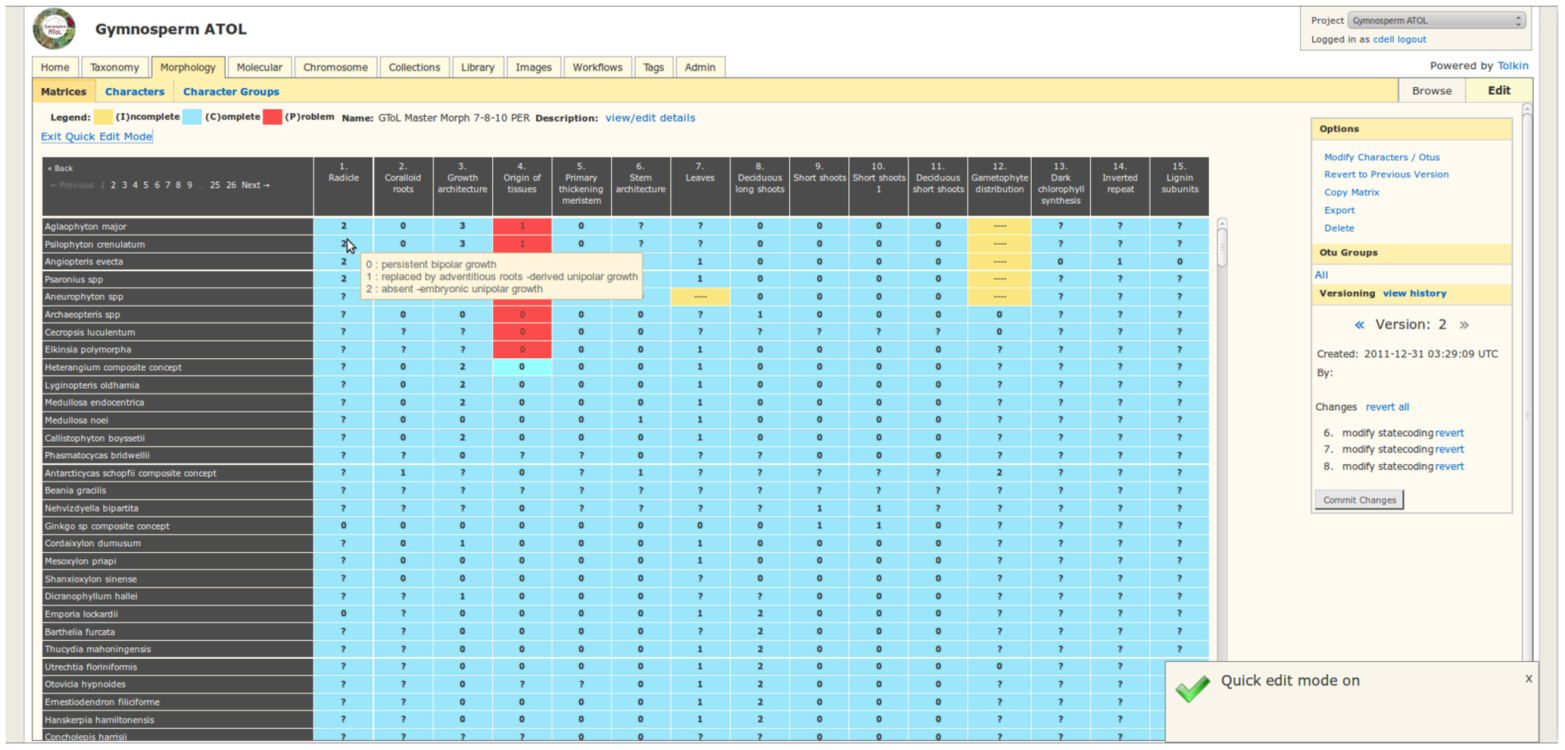
Morphology Module. Characters are defined in the ‘Characters’ tab and can be scored directly in each cell of the matrix. Characters can be grouped together and assigned to informal groups (‘Character groups’). OTU groups from the taxonomy module and character groups can be imported into a matrix for viewing, scoring and general editing.

## Methods

### Design and implementation

The development of TOLKIN ver. 2 started in 2007 using a Ruby-on-Rails (RoR) framework. A previous version was prototyped using PHP. Ruby-on-Rails is a model view controller (MVC) framework intended to facilitate rapid development and deployment of web-based resources that interface with database management systems. The design team has taken a modular approach to the architecture so that novel or unanticipated needs of the user community can be added. The underlying software libraries, and their dependencies, on which the TOLKIN infrastructure is built are to every extent possible, free and open source. These include the Linux operating system, PostgreSQL database, PostGIS and dependent libraries, Ruby, Ruby on Rails, BioRuby, Ajax, and a host of additional Javascript libraries. TOLKIN, itself, has been developed as a centrally managed collaboration platform rather than as a package for distribution, but the code is portable and the mature code will be made available through an open source repository. TOLKIN full technical description is available at: http://www.tolkin.org/technical.html.

**Figure 6:**
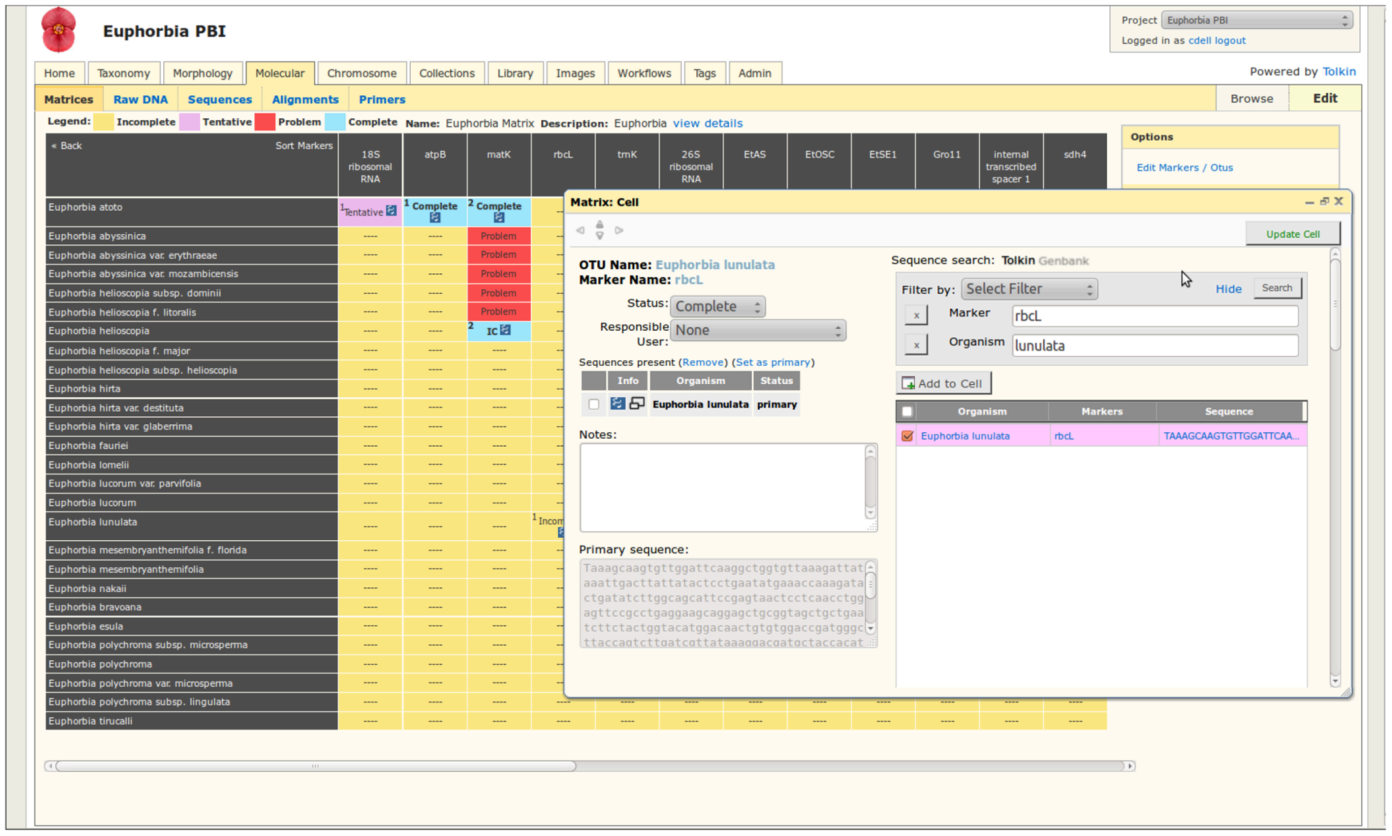
Molecular module. Data can be entered in each tab or through the matrix view. Each cell is interactive and linked to GenBank if a number is provided or when sequences are directly imported from GenBank. Data is exported into fasta files. Alignments and primer information can be stored for sharing and future repurposing.

Development efforts have generally focused on ease of use, flexibility in setting public access, capability to bulk import data from resources such as GenBank and to integrate with other services and stand-alone software through common formats, including Nexus, NeXML, Darwin Core, and spreadsheets.

**Figure 7:**
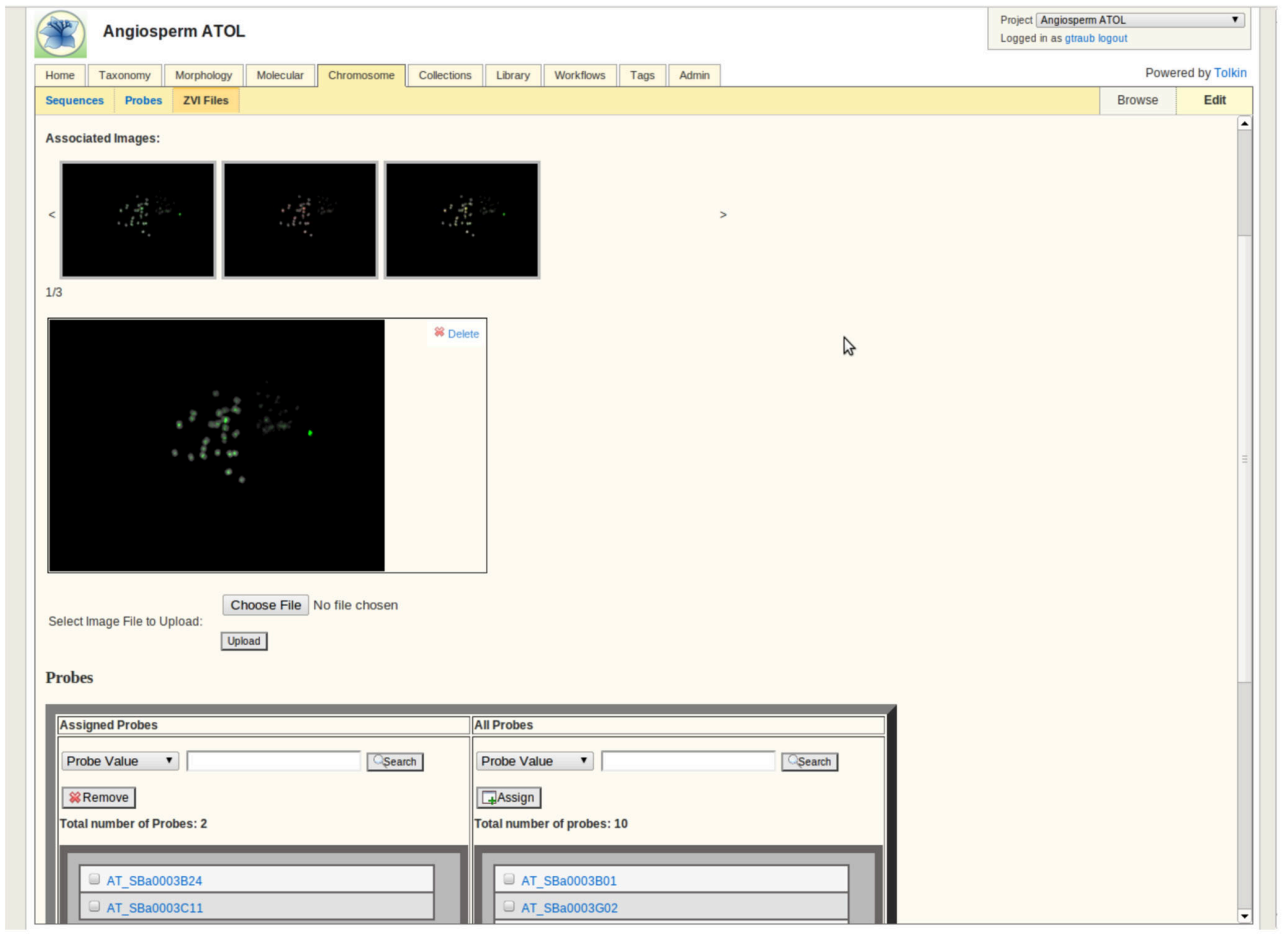
Chromosome Module. This module allows storing of information regarding chromosome probes, source sequences, and ZVI files containing images.

Behind the user interfaces, the RoR framework connects to a PostgreSQL backend database with a schema consisting of ca. 120 relational tables (Figure 2; see Results). On the front end, TOLKIN development keeps up with the latest web standards and popular libraries like jQuery in order to provide a modern interactive interface for the users.

**Figure 8:**
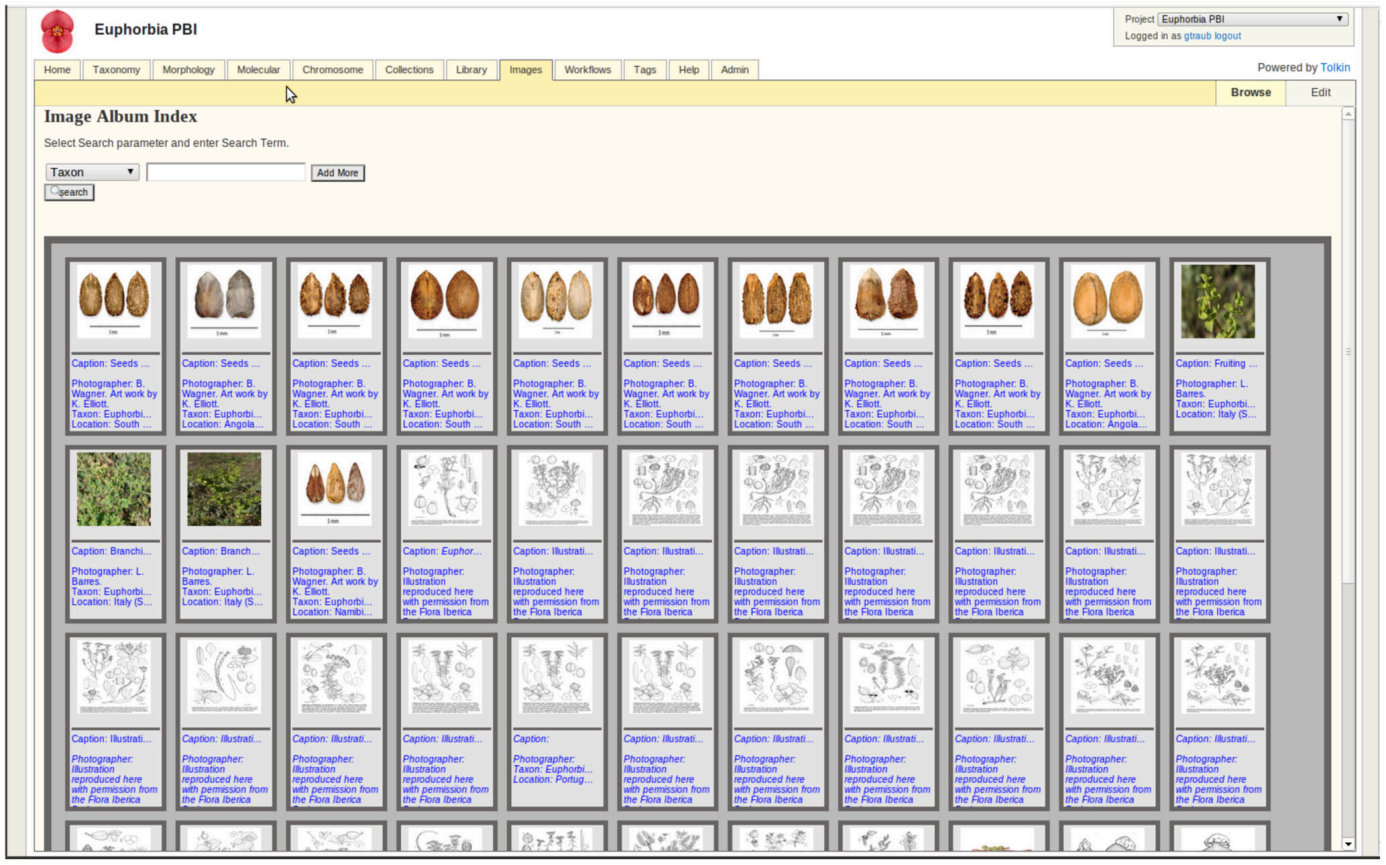
Image Gallery. A list of stored images is displayed. These can be searched based on different parameters and are linked across all modules.

The use of popular open source software like Ruby-on-Rails and jQuery helps TOLKIN comply with web publishing standards and ensures that much of the code behind it has been vetted by a large community of developers. This helps ease the development time needed to integrate and update the TOLKIN code.

An important set of features in TOLKIN involves batch processing or bulk data upload or acquisition. Users require the ability to import data from spreadsheets, collection databases, GenBank, and bibliographic management systems, and have collective access to shared resources. Access and permissions are managed so that individual collaborators have view, edit, or delete access depending on who owns the data records, and can constrain the project scope and levels of collaboration.

**Figure 9:**
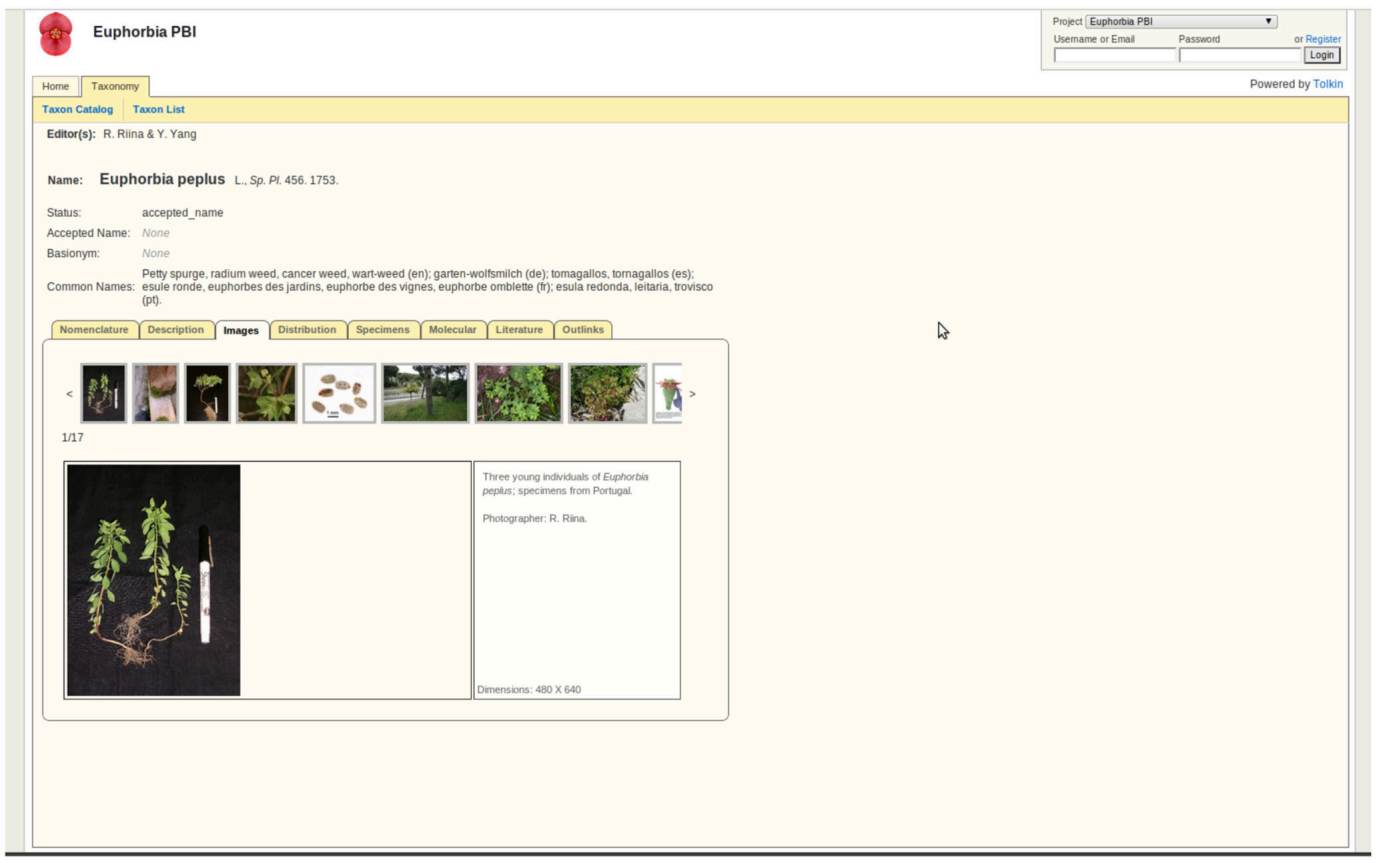
Taxon Pages. These pages are automatically generated based on information provided by users. Clicking on different tabs will show all data stored in each of the individual modules. Pages are publicly accessed, can be exported to other services and/or linked to project websites.

## Results

TOLKIN serves as a project information management system for collaborative efforts involving biodiversity research where integration of data between research laboratories is critical. Data management strategies are focused around day-to-day research use of taxonomic, molecular, morphological and bibliographic information. Taxon pages for public access are automatically generated to include user-selected information pertinent to any taxon at the level of species and/or clades. The information served to the community is based on data stored in any of TOLKIN modules and it is automatically updated as new data is acquired or modified by users. Additionally, publicly available information is ported through an automated export mechanism to resources such as Encyclopedia of Life (www.eol.org). This mechanism generates the required XML output file that EOL can ingest whenever users are willing to serve their data through this service.

**Figure 10:**
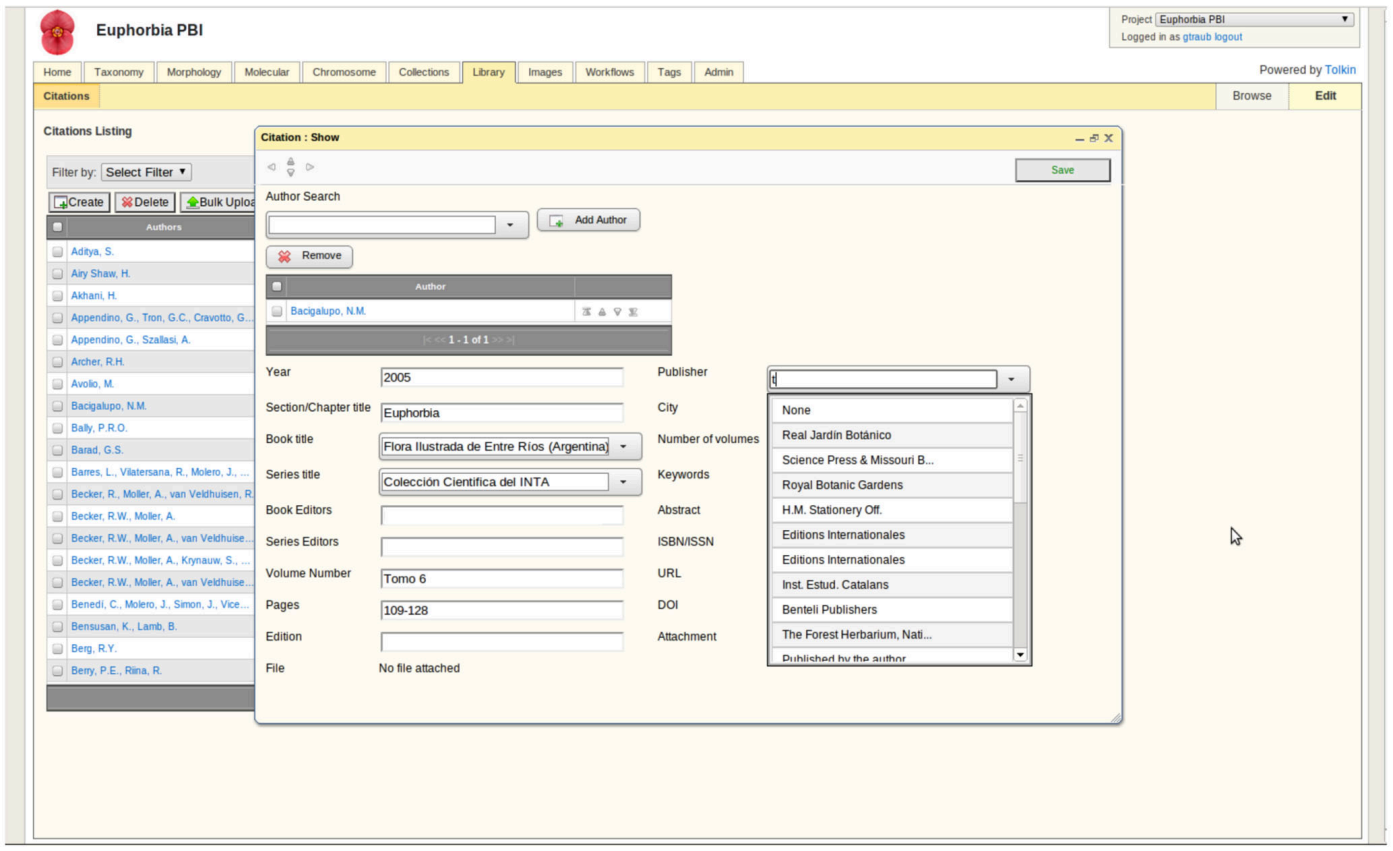
Library Module. Citation information is stored, view, and linked to data across all modules.

In general, data are handled in forms familiar to the practicing systematists, as specimen collections, morphological characters, genetic markers and operational taxonomic units (OTUs). The ability to view, link, and manage data records across various modules has been an overarching development goal in TOLKIN.

Figure 2 summarizes TOLKIN database schema, showing relationships of modules that support taxonomy, molecular and chromosome data, morphology, biological collections, images, and bibliography.

### TOLKIN modules

#### Taxonomy module (Figure 3)

Taxonomic data can be queried, browsed, and managed through tabular views and a hierarchical tree-based format with support for synonymies and nomenclatural annotations. The methods used to store taxa are independent of taxonomic rank and based on parent child relationships. Taxon ranks are stored as attributes, still maintaining the capability to search or view taxa by their rank. Every taxon has a parent, down to the root, and children, other than the terminals. Users are able to move branches in the taxonomic tree to reflect phylogenetic knowledge or they can choose to organize the tree according to alternative systems (e.g., alphabetically). Taxon names are associated with a code of nomenclature [e.g., ICZN, CPN, or PhyloCode] and carry attribute flags based on their synonymy to other entities and nomenclatural status.

#### Collections module (Figure 4)

TOLKIN handles collections as a critical means of vouchering the molecular and morphological data. Researchers typically source collections data from a variety of institutional sources. These, of course, are the primary custodians of the physical and digital voucher data, and TOLKIN serves as a project level aggregator of voucher data. In the case where institutions have not yet digitized their records, TOLKIN becomes the initial digital source, and plays a role in adding value to these data by maintaining provenance about links to related data, such as tissue samples and DNA extractions. Alternatively, in the Angiosperm AToL project, much of the collections data was sourced through the University of Florida Herbarium (FLAS), and TOLKIN simply stored a URI back to the primary resource. Projects such as Filtered Push [14] are currently developing methods of pushing value-added data back to primary custodians. Data can be imported from tabular format and spreadsheets, with automated mapping to commonly used column headings (e.g., Darwin Core).

#### Morphology module (Figure 5)

Data management is oriented around OTUs and characters, and matrices that combine the former, as reflected in standard practice in systematic biology. The interface supports matrix views, sub-setting, combining, and versioning of datasets, greatly simplifying tasks necessary to repeat experiments, re-use data and provide easier accessibility. OTUs have operational flexibility and can represent a published taxon, an informal name, or an individual collection. Characters are defined by description and character state values. Users can link documenting images, media, and collections to characters, character states, and OTUs.

Projects typically maintain a master matrix and individual views maintained by researchers or teams. Data is versioned at several levels, enabling users to roll back to a snapshot that was generated automatically or by choice. Version snapshots are taken whenever multiple matrices are merged or segregated, or when data values change. When matrices are merged, validation checks resolve conflicts that may have occurred while versions were modified. Users are asked to select which source they want to provide default values.

Users are able to import/export matrices to and from Nexus files and support for other file formats such as NeXML will soon be implemented. Additionally, user selected OTUs and characters can be exported from the matrix view.

#### Molecular module (Figure 6)

Data is managed around Matrices, DNA information, sequences, alignments, and primers. Like the morphology module, the molecular module employs OTUs, but the matrix columns are oriented around genetic markers as an operational unit. Therefore, each cell of the matrix represents one or more digital objects containing a sequence for a given OTU and marker. Each matrix cell displays status and metadata noting responsible parties. Color-coding and mouse-overs are used to quickly denote status and key information so that collaborators are able to easily scan for complete data, missing data, etc. Users can store as many sequences per OTU/marker combination, but one is indicated as primary for use in analysis.

Sequences are stored as unaligned and can be added individually, in bulk from spreadsheets, or from GenBank. GenBank imports can be based on GenBank numbers, specific taxa or markers. In the import process, user validation is required to select the desired sequences to add. As sequences are used in multiple alignments, the alignments can be archived and linked to the individual source sequences. It is up to the users whether they want to save alignments for later reference.

#### Chromosome module (Figure 7)

This module is designed to manage data related to chromosome analyses, for example Fluorescence *In Situ* Hybridization (FISH). Information related to BAC (Bacterial artificial chromosome) probes and target sequences are linked to ZVI files with images. This module supports research related to constructing karyotypes and studying genome-wide changes and integrating next-generation sequencing data into molecular cytogenetics.

#### Image gallery (Figure 8)

The use of digital images to document collections and observations has skyrocketed, and will continue to increase. Beyond generic public repositories for photos (e.g., Flickr), MorphBank (www.morphbank.net) and MorphoBank [11] have goals similar to TOLKIN in providing researchers the ability to store and manage images together with the metadata and related data. TOLKIN's emphasis is on generating links between images (and other media) to related data records or matrices. The chromosome module is a good example of how chromosome images are connected to specific data files and source of molecular information.

#### Bibliography module (Figure 9)

This module provides a shared framework for access to bibliographic citations that users maintain for the project and links to data in any of the other modules. Common bibliographic formats are supported (e.g., Endnote, Bibtext) for import.

#### Taxon pages (Figure 10)

Taxon/clade page for public dissemination are generated from the same data that users store and manage for their research. Usually, formats are similar to those used by EOL and Wikipedia. However, users can provide their preferred page format that is implemented for public dissemination of data. More commonly, taxon pages include core taxonomic data, morphological descriptions, images, distribution data, maps, specimens examined, molecular information (e.g., available sequences and sequenced taxa), etc. Enhanced interactive mapping capability that can display point occurrences of collections is implemented, and we will soon support “What's in my backyard?" queries (a geospatial query), where a taxon or collections list is returned based on proximity to a mouse click on the map. These types of queries can automatically generate a species inventory list, or e-flora, for a selected geographic region (e.g., country, state/province, county, national park) or at a specified radius from a point.

As projects mature or are completed, data can finally be pushed to long-term archival repositories. When users are ready to publicly serve their original data (e.g., images), they are encouraged to select or state the type of license under which they wish to release it.

A number of published studies demonstrate the use of TOLKIN to manage datasets for large phylogenetic analyses, data syntheses and taxonomic treatments [15]–[19].

## Discussion

There are a number of capabilities and priorities that have guided the development of TOLKIN. These include 1) handling a combination of molecular, morphological, collections, and taxonomic data not present in similar resources; 2) providing research teams from distant labs with the ability to collaborate on large biodiversity datasets in real-time; 3) maintaining provenance and versioning of molecular and morphological data; and 4) improving capabilities to synthesize data from multiple sources and thus facilitate porting data produced within projects to long-term, archival repositories (e.g., GenBank).

Researchers working individually have been well served by single-user desktop tools, up to a point. Collections databases, whether publicly (e.g., Specify) or commercially (e.g., EMu) funded are well adopted and used by museums and academic institutions that maintain collections and researchers that use those data for geospatial modeling, documenting monographs and revisions, and for managing taxonomic and nomenclatural information. Tools for descriptive data and key generation (e.g., Lucid) serve a separate purpose, as do web resources such as MorphBank and MorphoBank. TOLKIN does not perform the same tasks, but having the ability to integrate across those research domains is an infrastructure need that can be expected to grow.

As systematics, and biodiversity research in general, has become increasingly interdisciplinary and collaborative, tasks and responsibilities are divided among collaborators. The way data is shared becomes increasingly important to the repeatability of the analysis. The phalanx of single-user desktop tools has not kept up with this need. TOLKIN addresses data sharing through a centralized resource where users can perform essential functions of adding, editing, organizing, and integrating batch imports and exports in a way that sharing spreadsheets and other files, where versions become out of sync, does not.

These functions within TOLKIN are aimed at managing active projects, so that when these activities have run their course or users are ready to push data to archival resources such as Dryad and TreeBASE, they can do so. Users need to be able to maintain the provenance and repeatability of their analytical experiments (e.g., alignment, tree inference), and TOLKIN supports this requirement in a web-based multiuser environment. Essentially, collaborators are able to create master matrices that can become quite large in OTUs and characters/markers. Each user can generate subsets of the master and repurpose it for alternative or additional modifications; subsets can later on be re-integrated with the master file. As a whole, teams can view, manage, and track individual responsibilities for portions or the full dataset.

TOLKIN was not designed as a permanent repository, but the capability to push data out to permanent repositories such as GenBank and EOL is seen as essential. Unfortunately, with GenBank, this is still problematic, as NCBI does not maintain a stable easy-to-use set of web services for sequence deposit.

The management of matrix-based data is ubiquitous within the science community, and is specifically applied in both morphological and molecular data sets. TOLKIN provides a significant contribution not just in supporting shared editing, but also in maintaining views and control over layers of matrix-based data. This can be understood as analogous to a spreadsheet with embedded hyperlinks, where clicking on a cell exposes all the metadata about the content of that cell, including alternative and currently active values (e.g., observations or sequences made at different times). Within each cell, users can drill down through versioned layers of the matrix.

TOLKIN fills a need for managing biodiversity data at multiple levels, where users can describe objects at an atomic level (e.g., the sequences and primers related to a specific DNA extraction), or collectively as a dataset (e.g., the multiple sequences aligned and ready for analysis). Users can add, and manage sequences individually or import them in bulk from resources like GenBank. Data aggregation functions such as data import, and versioning are supported similarly. Users are able to change or designate a primary, or current, version of a sequence to be used in a particular analysis, and take a snapshot of a version of a matrix, modify it, run a new analysis, and if necessary, roll back to a previous version of the matrix. Instead of sharing spreadsheets or files, and having multiple versions floating around in email archives or file systems, where they often get out of sync, collaborators can see what everyone else is doing. Research teams are also able to designate who created and who is managing certain data elements. As collaboration and large data sets are becoming the norm in systematic biology, information infrastructure that is capable of supporting this growth must also grow and be usable by those who are not necessarily trained in informatics or computational sciences.

### Availability and Future Directions

Currently, TOLKIN is managing a number of collaborative projects funded by the National Science Foundation. As a server and service based platform, rather than a desktop application, there is an element of infrastructure maintenance that need to be addressed in terms of minimal support required for the software to run, and data to be maintained and accessed. This includes server maintenance, administration, software updates, and support to users. Beyond any potential to obtain funding for enhancements to TOLKIN, a subscription model is a viable alternative to offset maintenance costs. Subscribing collaborative projects that need data management and informatics support would benefit from previous investments and would not have to bear the burden of *de novo* informatics tooling.

Data management, analysis, and visualization of biological data are increasingly data and computationally intensive. This trend comes as a necessity through the advent and ubiquity of high-resolution and high-throughput data capture, e.g., next generation sequencing approaches. The availability and use of web services to provide access to computational capability for intensive and time-consuming analyses is increasingly common and biological research is becoming not just informatics-enabled, but informatics-dependent. Large datasets must be merged from various sources, sub-sampled, recombined, transformed and transmitted back and forth between desktop tools, web applications and servers. In practice, users must be technically adept and able to deal with heterogeneous inputs/outputs of web services. The use of scripting to pipeline data streams and different tools at various stages of analysis are essential. Pipelines can be effectively modeled as workflows, and desktop workflow software, e.g., Kepler [20], [21] and Taverna [22] among others, provide graphical environments with which to compose workflows according to the biological analysis procedure. Workflow software tools allow the integration of web services within analytical pipelines. The inputs/outputs of such web services running inside workflows usually tend to be simple-typed such as a piece of raw sequence or an object ID, which severely restricts the use of tools with more complex inputs/outputs in a workflow environment. As helpful as they are, existing workflow software remain hard to learn and/or challenging to master. TOLKIN is currently experimenting with complex workflows wrapped as web services and their streamlined deployment. A TOLKIN web service allows users to submit, run and manage a fully functioning abstract workflow composed of predefined tools. Abstract workflows are analytical pipelines whose components are specified by users but seamlessly designed and managed on the server side. Plans to build TOLKIN pipeline libraries are in place and these will initially include phylogenetic and population genetic analyses.

Finally, as data are exported and deposited into permanent repositories, there is a clear need to develop mechanisms for propagating data and metadata updates made in TOLKIN (e.g., taxonomic names). Although this is a desirable feature that will be a focus of future development, other projects such as BiSciCol (www.biscicol.blogspot.com) may also provide the alternative infrastructure to track changes of data and metadata stored in multiple repositories.
